# Exome Sequencing to Identify Novel Variants Associated with Secondary Amenorrhea and Premature Ovarian Insufficiency (POI) in Saudi Women

**DOI:** 10.3390/biomedicines12040785

**Published:** 2024-04-03

**Authors:** Ahmed M. Almatrafi, Ali M. Hibshi, Sulman Basit

**Affiliations:** 1 Department of Biology, College of Science, Taibah University, Al Madinah Al Munawarah 42353, Saudi Arabia; 2 Department of Obstetrics & Gynecology, King Sulman Medical City-Madinah Maternity and Children Hospital, Al Madinah Al Munawarah 42319, Saudi Arabia; dr_ali_hibshi@hotmail.com; 3 Department of Basic Medical Sciences, College of Medicine, and Centre for Genetics and Inherited Diseases, Taibah University, Al Madinah Al Munawarah 42353, Saudi Arabia; sbasit.phd@gmail.com

**Keywords:** premature ovarian insufficiency, secondary amenorrhea, infertility, exome sequencing, genetics, mutations

## Abstract

Background and objectives: Post-pubertal disappearance of menstrual cycles (secondary amenorrhea) associated with premature follicular depletion is a heterogeneous condition. Patients with this disease have low levels of gonadal hormones and high levels of gonadotropins. It is one of the causes of female infertility and a strong genetic component is attributed as an underlying cause of this condition. Although variants in several genes have been associated with the condition, the cause of the disease remains undetermined in the vast majority of cases. Methodology and Materials: Ten Saudi married women experiencing secondary amenorrhea were referred to a center for genetics and inherited diseases for molecular investigation. A family-based study design was used. Intensive clinical examinations, including pelvic ultra-sonography (U/S) and biochemical evaluations, were carried out. Karyotypes were normal in all cases and polycystic ovarian syndrome (PCOS) was excluded by using Rotterdam consensus criteria. Patients’ DNA samples were whole-exome sequenced (WES). Bidirectional Sanger sequencing was then utilized to validate the identified candidate variants. The pathogenicity of detected variants was predicted using several types of bioinformatics software. Results: Most of the patients have a normal uterus with poor ovarian reserves. Exome sequence data analysis identified candidate variants in genes associated with POI in 60% of cases. Novel variants were identified in *HS6ST1*, *MEIOB*, *GDF9*, and *BNC1* in POI-associated genes. Moreover, a homozygous variant was also identified in the *MMRN1* gene. Interestingly, mutations in *MMRN1* have never been associated with any human disease. The variants identified in this study were not present in 125 healthy Saudi individuals. Conclusions: WES is a powerful tool to identify the underlying variants in genetically heterogeneous diseases like secondary amenorrhea and POI. In this study, we identified six novel variants and expanded the genotype continuum of POI. Unravelling the genetic landscape of POI will help in genetic counselling, management, and early intervention.

## 1. Introduction

Secondary amenorrhea is the cessation of the menstrual cycle in women before the age of 40. Secondary amenorrhea causes premature ovarian insufficiency (POI) and, subsequently, infertility [1,2,3]. Thus, POI is characterized by the cessation of menstruation and the onset of menopause in women under the age of 40, leading to depletion or dysfunction of ovarian follicles [4,5]. The clinical manifestations of POI vary significantly among patients [6]. These symptoms may include primary amenorrhea (PA) or secondary amenorrhea (SA), hypoestrogenism, hypergonadotropinism, infertility or subfertility, and other menopause-related issues such as vaginal dryness, night sweats, dyspareunia, reduced sexual desire, and hot flushes [7,8]. The prevalence of POI has been estimated to be around 1 in 100 women of childbearing age [9].

The etiology of POI is highly diverse, and the primary cause remains unknown in 60–70% of cases [1,4]. Various factors have been associated with the development of POI, including metabolic syndromes, autoimmune factors, radiotherapy, chemotherapy, ovarian or pelvic surgery, infections, and genetic defects [10,11].

Although cases of POI can occur sporadically, familial cases contribute up to 31% of instances [5,6,11]. Abnormalities in the structure and number of the X chromosome have been found in 13% of cases [12]. Furthermore, the premutation of the *FMR1* gene has been detected in 6% of POI cases [13]. Pathogenic variants in various genes located on either autosomes or the X chromosome have been reported in multiple cases. Defects in genes related to mitochondrial functions and non-coding RNAs have also been implicated as potential risk factors for POI [11,12,13,14,15,16].

In recent years, advancements in high-throughput sequencing technologies have significantly enhanced our understanding of the pathogenesis of POI. To date, mutations in more than 90 genes have been identified as an underlying cause of POI, either in isolated cases or as part of syndromic conditions [14,15,16]. A recent large-scale study utilizing whole-exome sequencing (WES) in POI patients identified novel pathogenic variants in 20 genes involved in various aspects of ovarian function, including ovulation, meiosis, folliculogenesis, and gonadogenesis [17]. Although several POI candidate genes have been identified, the cause of POI remains undetermined in the vast majority of cases. Furthermore, a limited number of studies have been conducted on POI patients from the Saudi Arabian population to investigate the underlying genetic causes. Therefore, it is crucial to identify the molecular players underlying POI to explore potential therapeutic targets such as in vitro activation, as well as to provide guidance for genetic counseling or pregnancy planning [18]. The aim of this study was to identify the genetic defects among Saudi women presenting with secondary amenorrhea, POI, and infertility.

## 2. Materials and Methods

### 2.1. Recruitment of POI Patients/Controls and Ethical Approval

The study design used herein is a family-based genetic study. We investigated 10 females from various Saudi families who presented to the infertility clinic of Madinah Maternity and Children Hospital (MMCH), King Salman bin Abdul Aziz Medical City-KSAM (Al Madinah Al Munawarah, Saudi Arabia), with the chief complaint of secondary amenorrhea and infertility. Moreover, 125 healthy fertile female individuals without any family history of amenorrhea, PCOS, and POI were also included as controls. All participants provided informed consent and completed family history forms. The study was approved by the institutional review board of Taibah University (TUCDREC/27032021) and MMCH (IRB147-2021). Patients were provided with a written consent form in Arabic, and the study’s goals were explained to them before they signed the form. The study was conducted according to ethical guidelines established by the committee.

### 2.2. POI Selection Criteria

All 10 patients were diagnosed as having POI. The diagnostic criteria employed in this study for POI adhered to the guidelines outlined by the European Society of Human Reproduction and Embryology (ESHRE). According to these guidelines, a diagnosis of POI required the presence of oligo/amenorrhea lasting a minimum of 4 months along with elevated levels of FSH (>25 IU/L ) on two separate occasions, with an interval of more than 4 weeks, prior to the age of 40. To ensure accurate diagnosis, each participant underwent pelvic ultrasound, a comprehensive evaluation of their medical history and family pedigree, and chromosomal analysis. Patients with a history of chemotherapy, radiotherapy, ovarian surgery, chromosomal abnormalities, or autoimmune disorders were excluded from the study to minimize potential confounding factors.

All individuals included in this study experienced secondary amenorrhea (SA), indicating that they had previously undergone at least one spontaneous menstrual cycle. The age range of the recruited patients was 25 to 40 years. All participants self-identified as female, and their biological sex was confirmed through ultrasonography and karyotype analysis.

### 2.3. Biochemical Profile and Hormonal Analysis

A complete blood count, biochemical profile, and hormonal analysis were conducted for all participants at MMCH in Madinah. Hormonal determination was carried out on day 3 of the cycle. Hormonal analysis included measurements of estradiol (E2), prolactin (PRL), thyroid-stimulating hormone (TSH), follicle-stimulating hormone (FSH), and luteinizing hormone (LH).

### 2.4. Genomic DNA Extraction and Spectrophotometry

A total of 3 mL of peripheral blood was collected from each participant in an ethylenediaminetetraacetic acid (EDTA) tube for genetic evaluation. Genomic DNA was subsequently extracted from the collected blood samples using the Qiagen QiaAmp DNA mini kit (Qiagen, Manchester UK), following the manufacturer’s guidelines. The quality and purity of extracted DNA were measured using a Qubit fluorometer and a Nanodrop-1000 spectrophotometer (Thermo Fisher Scientific, Waltham, MA, USA).

### 2.5. Genomic Library Preparation and Whole-Exome Sequencing (WES)

Ten genomic DNA samples from POI patients underwent WES following a previously described procedure [19,20]. Exome sequencing was carried out using the Illumina Hiseq2000 machine, and DNA libraries were prepared using the SureSelect Kit from Agilent (Santa Clara, CA, USA). The BaseSpace online tool (Illumina, San Diego, CA, USA) was utilized for standard filtration of the variant calling files (VCFs) of all participants. Consequently, only homozygous and compound heterozygous variants associated with POI present in affected participants were considered for further analysis. The identified variants were subsequently classified according to the guidelines provided by the American College of Medical Genetics and Genomics (ACMG).

### 2.6. Prediction of the Pathogenicity of Variants

The exome sequence data were thoroughly analyzed, and the frequency of suspected homozygous or biallelic variants was validated against various public genomic databases such as Exome Variant Server (https://bio.tools/exome_variant_server/ accessed on 2 January 2024), dbSNP (https://www.ncbi.nlm.nih.gov/snp/ accessed on 4 January 2024), gnomAD (https://gnomad.broadinstitute.org/ accessed on 4 January 2024), and the 1000 Genomes browser (https://www.ncbi.nlm.nih.gov/variation/tools/1000genomes/ accessed on 8 January 2024)] and the in-house WES data of 125 healthy Saudi individuals from different ethnic groups.

In addition, the pathogenicity of each identified variant and its effect on the overall protein structure was assessed using multiple bioinformatic tools. These online bioinformatic tools include VarSome (https://varsome.com/ accessed on 10 January 2024), FATHMM (http://fanthmm.biocompute.org.uk/ accessed on 12 January 2024), CADD (https://cadd.gs.washington.edu/ accessed on 12 January 2024), PolyPhen-2 (http://genetics.bwh.harvard.edu/pph2/ accessed on 14 January 2024), PredictProtein (https://predictprotein.org/ accessed on 14 January 2024), Mutation Assessor (http://mutationassessor.org/ accessed on 14 January 2024), SIFT (http://sift.bii.a-star.edu.sg/ accessed on 15 January 2024), and MutationTaster (http://www.mutationtaster.Org/ accessed on 15 January 2024).

### 2.7. Design of Specific Primers and Validation of Variants through Sanger Sequencing

The candidate variants identified in affected individuals were analyzed using bidirectional Sanger sequencing to confirm the findings, following a previous protocol described elsewhere [19,20]. Primer 3 (Version 04.0) software was employed to design specific primers for the candidate gene, and the Ensembl genome browser was utilized to download the reference sequences of the target genes. The resulting sequencing reads were aligned with the reference sequence using BIOEDIT software version 6.0.7 to confirm the presence of variants.

### 2.8. In Silico Analysis

NCBI HomoloGene (http://www.ncbi.nlm.gov/homologene/ accessed on 2 February 2024) was utilized to determine the conservation of the amino acid sequence of the identified genes throughout different species. UNIPROT (https://www.uniprot.org accessed on 3 February 2024) was employed to map the mutated variants to the specific protein domain. For the investigation of protein–protein interaction networks, the STRING website (version 12) was used.

## 3. Results

### 3.1. Clinical Characteristics and Hormonal Analysis Evaluation

A total of 10 index patients diagnosed with POI and infertility were enrolled in this study from February 2021 to February 2023. Table 1 illustrates the demographic data of the patients. Among the participants, 50% (5 out of 10) had a positive family history of infertility or POI. All of the patients were married, with an average marriage duration of 8.8 years. The body mass index (BMI) of the patients ranged from 23 to 31.9, with an average of 26.4. All of the participating patients were of Arab ethnicity.

In terms of clinical characteristics, all patients in the study experienced secondary amenorrhea, and 70% (7 out of 10) exhibited mood disturbance. Additionally, 40% (4 out of 10) reported changes in their sleep cycle, while 60% complained of dyspareunia due to atrophic vaginitis. Approximately 80% of the patients sought medical attention for primary infertility, while 20% sought help for secondary infertility.

Rotterdam consensus criteria were used to rule out PCOS in all patients; however, poor ovarian reserves were observed in 90% of patients (Table 2). The hormonal serum analysis investigation showed that the median range of estradiol (E2) was 32.64 pg/mL (N:12–166 pg/mL), that of prolactin (PRL) was 377.32 mU/L (N: <25 ug/L), that of thyroid-stimulating hormone (TSH) was 3.64 m lU/L (N: 0.4–2.5 m lU/L), that of follicle-stimulating hormone (FSH) was 46.288 lU/L (N: 1.5–12.4 lU/L), and that of luteinizing hormone (LH) was 723.03 lU/L (N: 5–25 lU/L).

### 3.2. POI-Associated Variants Identified in Patients

**Figure 1:**
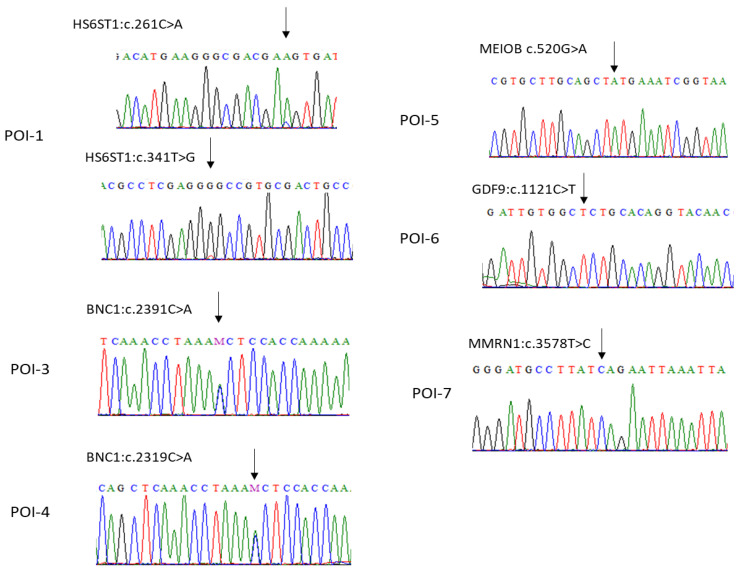
Sanger sequencing validation of identified pathogenic variants among POI patients. Patient POI-1 harbored two compound homozygous variants in the *HS6ST1* gene (c.261C>A and c.341T>G). POI-3 and POI-4 had heterozygous variants (c.2319C>A) in the *BNC1* gene. POI-5 inherited a homozygous variant (c.520G>A) in the *MEIOB* gene. Patient POI-6 carried a missense pathogenic variant in the GDF9 gene (c.1121C>T) while POI-7 harbored a pathogenic homozygous variant (c.3578T>C) in the *MMRN1* gene.

To understand the genetic factors involved in the development of POI in the selected patients, we performed WES on DNA samples obtained from 10 individuals with POI. The exome sequencing reads had coverages exceeding 100×. We selectively considered variants for our analysis, filtering out those with a minor allele frequency (MAF) of more than 1% in the 1000 G, gnomAD, ESP6500 and dbSNP databases. Subsequently, we applied a filtering process to the remaining variants with an MAF < 1%, focusing on specific candidate genes known to be associated with POI. We detected a total of six variants in five genes, including homozygous variants in *HS6ST1*, *MEIOB*, and *GDF9*. Additionally, a heterozygous variant in *BNC1* was found in two patients (Table 3). None of these variants were identified in the WES data obtained from 125 healthy Saudi individuals of various ethnic backgrounds from our in-house dataset. The exome data were also screened using an unbiased and hypothesis-free approach. In this case, we looked for variants of interest in all genes. A damaging homozygous variant was identified in the *MMRN1* gene. To validate the identified variants, bidirectional Sanger sequencing was performed for all identified variants (Figure 1). In the case of patient POI-1, two compound homozygous variants were found in the *HS6ST1* gene (NM_004807.3; c.261C>A; p. Asp87Glu and NM_004807.3; c.341T>G; p. Val114Gly). Conversely, POI-3 and POI-4 had heterozygous variants (NM_001717.4; c.2319C>A; p. Asn773Lys) in the *BNC1* gene. POI-5 harbored a homozygous variant (NM_001163560.3; c.520G>A; p. Val174Met) in the *MEIOB* gene. Additionally, patient POI-6 carried a missense pathogenic variant in the *GDF9* gene (NM_005260.7; c.1121C>T; p. Pro374Leu) while POI-7 harbored a rare homozygous variant (NM_007351.3; c.3578T>C; p. Leu1193Ser) in the *MMRN1* gene. Furthermore, no variant associated with POI was identified in patients POI-2, POI-8, POI-9, and POI-10 after comprehensive analysis of the patients’ exome data.

To better understand the impact of the identified variants on the development of and predisposition to POI, various bioinformatics tools were utilized such as Revel, SIFT, MutationTaster, CADD, FATHMM, DANN, Mutation assessor, and PROVEAN to estimate the pathogenicity score of each identified variant (Table 3). All identified variants were novel missense variants and classified as variants of unknown significance (VUS) based on ACMG classification.

*HS6ST1*, *BNC1*, *MEIOB*, and *GDF9* have been well documented to be associated with POI and are known to play crucial roles in essential biological processes such as the regulation of oocyte maturation, homologous recombination, and ovarian folliculogenesis. Interestingly, we observed pathogenic variants in one gene not previously reported to be associated with POI; this gene is *MMRN1*.

### 3.3. In Silico Analysis

**Figure 2:**
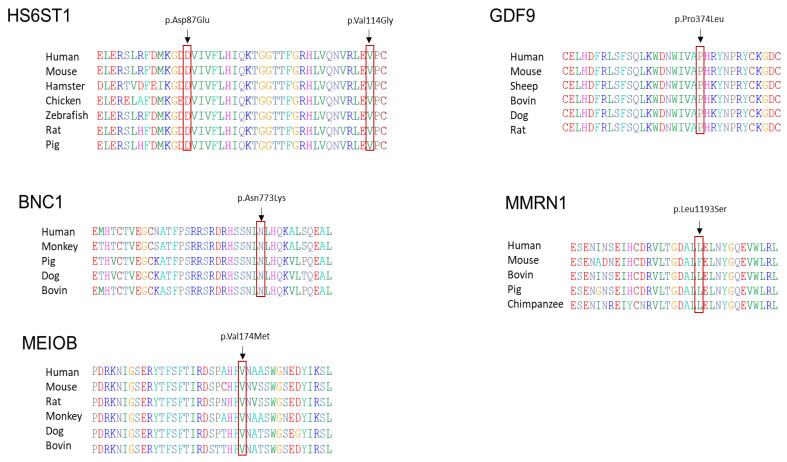
The analysis of protein conservations. All identified amino acids (p. Asp87Glu and p. Val114Gly) in HS6ST1, (p. Asn773Lys) BNC1, (p. Val174Met) MEIOB, (p. Pro374Leu) GDF9, and (p. Leu1193Ser) MMRN1 showed a high degree of conservation across a wide range of species, including mice, pigs, bovines, dogs, monkeys, and rats.

To assess the impact of the identified variants on protein stability and function, manual mapping was conducted to associate each pathogenic variant with its corresponding protein. This mapping was performed using the NCBI Nucleotide database and UNIPROT software (https://www.uniprot.org/ accessed on 3 February 2024). The analysis revealed that the amino acid sequences of HS6ST1 (p. Asp87Glu and p. Val114Gly), BNC1 (p. Asn773Lys), MEIOB (p. Val174Met), GDF9 (p. Pro374Leu), and MMRN1 (p. Leu1193Ser) are highly conserved across various species, including mice, pigs, bovines, dogs, monkeys, and rats (Figure 2).

### 3.4. Protein Interaction Network of Candidate POI Genes

**Figure 3:**
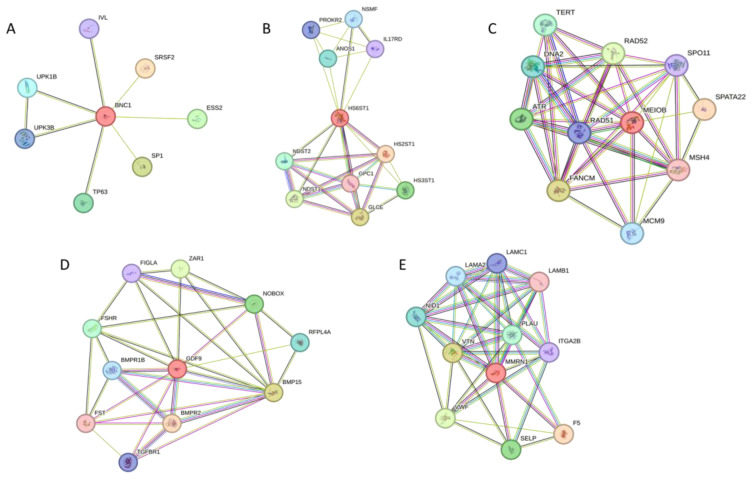
Protein–protein interaction network analysis of the identified genes associated with POI. (**A**) *BNC1* analysis network, (**B**) *HS6ST1*, (**C**) *MEIOB*, (**D**) *GDF9*, and (**E**) *MMRN1*.

Protein–protein interaction analysis using STRING tools revealed significant genetic and physical interactions of HS6ST1 with various proteins, including ANOS1, IL17RD, NSMF, and PROKR2 (Figure 3). These proteins are associated with the development of hypogonadotropic hypogonadism and can contribute to Kallmann syndrome. MMRN1 displayed a high level of association with the coagulation factor V (F5) protein, which is involved in recurrent pregnancy loss. Another known POI-related gene, BNC1, exhibited strong interactions with other proteins associated with POI type 21, such as TP63. MEIOB, on the other hand, demonstrated strong interactions with proteins involved in meiotic recombination, including SPO11, RAD51, RAD52, MCM9, and MSH4. Additionally, the protein GDF9 showed significant genetic and physical interactions with BMP15, NOBOX, and FSHR, which play roles in folliculogenesis development and the regulation of oocyte function.

## 4. Discussion

In the current study, we recruited a total of 10 Saudi married women who had been clinically diagnosed with POI and infertility. WES was conducted on DNA samples to identify underlying pathogenic variants associated with the development of the disease. Four novel variants were identified in previously known genes associated with POI. Heparan sulfate 6-O-sulfotransferase 1 (HS6ST1) is a member of the heparan sulfate (HS) family of proteins, which are known to play crucial roles in a variety of cellular functions, including cellular morphology, migration, differentiation, proliferation, adhesion, and the maintenance of stem cells [21,22]. Mutations in HS6ST1 have been identified among different families with idiopathic hypogonadotropic hypogonadism (IHH) [23]. These patients typically exhibit several clinical phenotypes, including infertility and incomplete or absent puberty [23,24]. In the case of our patient (POI-1), two compound homozygous variants (c.261C>A and c.341T>G) were detected in the HS6ST1 gene. These variants have been found to be located in highly conserved regions across various species. Our findings provide further support for the results of a previous study in which the authors identified a chromosomal breakpoint in the HS6ST1 gene at the 2q21 region in one POI patient [25].

This study identified a variant of unknown significance in the zinc finger transcription factor Basonuclin 1 (BNC1) in two patients with primary infertility and poor ovarian reserve. BNC1 is a transcriptional factor which is highly expressed in the germline cells of both the ovary and testis [26]. It plays a regulatory role in oogenesis and folliculogenesis, specifically in the transcription of ribosomal RNA (rRNA). Studies on Bnc1 knockout mice have shown that the absence of Bnc1 leads to mice subfertility [26,27]. BNC1 haploinsufficiency has been documented as a cause of autosomal dominant POI [27]. In the current study, both patient POI-3 and patient POI-4 carry a heterozygous variant c.2319C>A in the BNC1 gene, and this is predicted as a variant of uncertain significance. Our results are supported by a recent finding stating that heterozygous mutations in BNC1 can lead to the development of POI [18].

Meiosis specific with OB-fold (MEIOB) is a meiosis-specific ssDNA-binding protein. It is mainly involved in the regulation of early meiotic prophase I and the formation of synapsis between homologous chromosomes [28]. Null mice of Meiob−/− fail to complete meiotic recombination due to the accumulation of unrepaired double-strand breaks (DSBs), which leads to infertility in both males and females [29,30]. Pathogenic variants in MEIOB were shown to cause POI and non-obstructive azoospermia (NOA) in a consanguineous family [31,32]. We identified a homozygous missense variant (c.520G>A; p. (Val174Met)) in the MEIOB gene in an infertile patient. This variant is located in the conserved region of MEIOB (OB-fold) that binds to single-stranded DNA/replication protein A1 (RPA1). This region plays an essential role in the direct interaction between MEIOB-SPATA22 and the RPA complex, which is crucial for the recruitment of DSBs [32]. The patient complains of primary infertility as well as POI.

The growth-differentiation factor 9 (GDF9) is predominantly expressed in oocytes and has a vital role in normal ovarian folliculogenesis as well as female fertility [33]. Yan et al. showed that mice with Gdf9−/− knockout are sterile due to impaired folliculogenesis at the primary follicle stage [34]. Mutations in the GDF9 gene have been reported to cause POI and infertility among women from different ethnic groups [35,36,37]. Our patient (POI-6) carries a missense pathogenic variant in the GDF9 gene (c.1121C>T; (p. Pro374Leu)), and in silico analysis has shown that this region is highly conserved in various species. This patient suffers from primary infertility and irregular menstrual periods. However, pelvic ultrasound showed normal ovarian reserves.

Multimerin 1 (MMRN1) is a member of the EMILIN protein family that can form large, soluble, polymeric structures and specifically binds to factor V (FV). It is primarily found in storage granules within platelets and endothelial cells but is not typically detectable in normal plasma [38,39]. Male mice with knockout of the Mmrn1 gene displayed infertility alongside an increased number of large unstained cells (https://www.mousephenotype.org/data/genes/MGI:1918195 accessed on 5 February 2024). Our case POI-7 presents with POI and primary infertility and harbors a rare pathogenic homozygous variant (c.3578T>C) in the MMRN1 gene. A previous study showed that a homozygous variant (c.3454G>A) in the MMRN1 gene was associated with male infertility in a Saudi patient, supporting our novel finding of a potential role for the MMRN1 gene in POI and infertility among women [40]. Our index patient showed primary infertility and had poor ovarian reserves.

POI is a heterogeneous disease that can result from several factors, with sexual dysfunction being one of the major common symptoms among women with POI. The early onset of gonadal function loss is particularly distressing for young women and has wide-ranging effects on their reproductive ability, physical health, and mental well-being [41]. Hormone replacement therapy can reduce clinical symptoms among affected individuals; however, the effectiveness of these therapies for impaired reproductive function is limited [18].

Early detection of and intervention for POI plays an important role in managing the disease and its impact on female fertility. Identifying POI at an early stage permits proactive steps, such as fertility preservation methods. WES is an effective and affordable tool for the identification of pathogenic variants, particularly in unexplained POI cases or POI patients without obvious somatic anomalies [18]. Although we successfully identified the variant in 60% of cases, this study has some limitations, however. Large-scale studies with a huge number of samples are required to replicate the results of this study. Moreover, in vivo studies using animal models are needed to validate the variants identified in this study.

## 5. Conclusions

Our study involved the recruitment of 10 patients diagnosed with POI and infertility. We successfully identified pathogenic variants associated with POI in 60% (6/10) of our cases. This demonstrates the effectiveness of WES in elucidating the genetic causes of POI. The findings from our research highlight the potential of WES as a valuable tool in determining the genetic basis of POI. This knowledge can provide a theoretical foundation for early detection and intervention in individuals at risk of developing POI in the future.

## Figures and Tables

**Table 1:** Clinical characterization of affected patients with secondary amenorrhea and POI.

Patient No.	Age (Years)	Family History	Menopause Age (Years)	Married for (Years)	BMI (kg)	Mood Disturbance	Sleep Cycle Changing	Dyspareunia Due to Atrophic Vaginitis	Rule Out PCOS	Other Clinical Symptoms
POF-1	25	No	23	7	24	Yes	No	Yes	Yes	Secondary infertility
POF-2	27	Yes	25	8	31.9	No	No	No	Yes	Primary infertility
POF-3	30	No	29	15	27	Yes	No	No	Yes	Infertility
POF-4	40	No	38	2	23.4	No	No	No	Yes	Infertility
POF-5	35	Yes	32	12	31.56	No	No	No	Yes	Infertility
POF-6	27	No	25	6	24.4	Yes	Yes	Yes	Yes	Infertility and irregular last menstrual period
POF-7	29	Yes	26	8	24	Yes	No	Yes	Yes	Infertility
POF-8	36	No	35	10	31	Yes	Yes	Yes	Yes	Irregular cycle, infertility, and hypothyroidism
POF-9	35	Yes	33	14	24	Yes	Yes	Yes	Yes	Secondary infertility
POF-10	33	Yes	30	6	23	Yes	Yes	Yes	Yes	Infertility

**Table 2:** Hormonal analysis and pelvic ultrasound of affected individuals with POI.

Patient No.	Estradiol (pg/mL)	PRL (mU/L)	TSH (mlU/L)	FSH (lU/L)	LH (lU/L)	AMHng/mL	Pelvic Ultrasound
POF-1	40	106	4.6	77	15.38	Not performed	Normal uterus with poor ovarian reserves
POF-2	5	208.6	1.23	39.52	16.45	Not performed	Normal
POF-3	136.2	217.24	0.991	49.64	38.46	Not performed	Poor ovarian reserves
POF-4	16.51	173.8	1.79	54.51	21.3	0.010	Poor ovaries reserves
POF-5	15.25	694.77	7.68	44.78	25	Not performed	Normal ovaries
POF-6	18	227	4.5	60	13.18	0.05	Normal ovaries
POF-7	16.31	753.42	3.566	20.49	70.15	Not performed	Poor ovarian reserves
POF-8	47.13	248.4	5.19	12.94	3.43	Not performed	Poor ovarian reserves
POF-9	13	53	2.07	16.1	2.11	Not performed	Poor ovarian reserves
POF-10	19	1091	4.8	98	80	Not performed	Poor ovarian reserves

**Table 3:** In silico analysis of identified variants associated with POI found using whole-exome sequencing.

Patient No.	POF01	POF01	POF03/04	POF05	POF06	POF07
Gene	*HS6ST1*	*HS6ST1*	*BNC1*	*MEIOB*	*GDF9*	*MMRN1*
Chromosome	2	2	15	16	5	4
Nucleotide variant	c.261C>A	c.341T>G	c.2319C>A	c.520G>A	c.1121C>T	c.3578T>C
SNP	rs200979099	rs199993343	rs1453233120	rs1025418947	rs373477788	rs201798101
Protein variant	p.Asp87Glu	p.Val114Gly	p.Asn773Lys	p.Val174Met	p.Pro374Leu	p.Leu1193Ser
Zygosity	Homo	Homo	Hetero	Homo	Homo	Homo
Exon	1	1	5	6	2	8
gnomAD Freq	0.00941	0.0018	0.00000657	0.0000197	0.0000591	0.0000723
ACMG classification	VUS	VUS	VUS	VUS	VUS	VUS
Prediction bioinformatic tools						
SIFT	P	BM	P	U	P	U
Revel	P	U	B	U	P	P
PROVEAN	U	BM	U	U	PM	B
Mutation Taster	U	U	B	U	VUS	BM
CADD	25	21	2.584	26.3	27.4	27
Mutation assessor	P	BM	U	U	P	P
FATHMM	U	BM	B	BM	VUS	U
DANN	U	U	U	U	U	U

Abbreviation: Variant of uncertain significance (VUS); pathogenic (P); pathogenic moderate (PM); benign (B); benign moderate (BM); uncertain significance (U).

## Data Availability

Data are available from the authors upon request.
